# Management of pain in cancer patients – an update

**DOI:** 10.3332/ecancer.2024.1821

**Published:** 2024-12-12

**Authors:** María Laura Daud, Gustavo G De Simone

**Affiliations:** 1 Instituto Pallium Latinoamérica, Av Caseros 2061, Ciudad Autónoma de Buenos Aires C1264, Argentina; 2 Facultad de Medicina de la Universidad del Salvador, Av Córdoba1601, Ciudad Autónoma de Buenos Aires C1055AAG, Argentina; 3 Consejo de Ética en Medicina, Academia Nacional de Medicina de Buenos Aires, Av Gral. Las Heras 3092, Ciudad Autónoma de Buenos Aires C1425ASU, Argentina; 4 Programa Estar, Ministerio de Salud de la Ciudad de Buenos Aires, Av Medrano 350, Ciudad Autónoma de Buenos Aires C1179AAF, Argentina; a https://orcid.org/0000-0003-1113-7531; b https://orcid.org/0000-0003-2037-9997

**Keywords:** pain, cancer, palliative care, analgesics

## Abstract

Pain is one of the most detrimental symptoms exhibited by cancer patients, being an indication for opioid therapy in up to half of the patients receiving chemotherapy and even more for those with advanced cancer. This article aims to briefly overview current knowledge on cancer-related pain with a focus on assessment and new approaches and trends. We will also provide some insight on the lower- and middle-income countries context.

**Data sources:**

A narrative review of the literature was conducted including relevant guidelines and recommendations from scientific societies and WHO.

**Data summary:**

Data on the approach and assessment of cancer pain as well as current and novel approaches have been displayed with the help of tables and figures.

**Conclusion:**

Since the initial recommendations of the WHO analgesic ladder method, new insights have emerged. Scientific progress reaches its maximum social sense when populations and governments prioritise the value of relief and compassion, and concrete actions are implemented with the aim of relieving cancer pain.

## Introduction

Pain is one of the most frequent and feared symptoms in people with cancer. Evidence shows that 30% to 50% of cancer patients will experience moderate to severe pain. It may occur at all stages of the disease, although it may increase in intensity and frequency if cancer progresses. Over one third of patients may suffer pain even after curative treatment [1–3]. Unrelieved pain can interfere with all aspects of daily living [4, 5] with negative influences on patients’ clinical outcomes, wellbeing and satisfaction [6, 7]. On the contrary, adequate pain management improves health-related quality of life (HRQL), reduces unexpected health resources use and improves adherence to cancer treatments [8]. Recent data suggest that pain control may influence cancer survival rates as well [9].

It is demonstrated that a proper level of analgesia may be achieved in over 90% of cancer patients within 14 days of current pharmacological approaches [10, 11]. During the last decades, research has led to major contributions on understanding pain pathophysiology and management. Despite these, cancer pain is still highly prevalent [1, 12, 13] and undertreated in one over two patients, with a significant detrimental impact on physical, psychological and social functioning [14].

Several barriers to effective pain control have been pointed out, related to pain quality, professionals or patients’ attitudes, training, cultural or economic factors and regulatory issues [1, 15–17]. Acknowledging them is crucial to encourage overcoming policies and actions that would better respond to patients’ needs.

Pain relief is an ethical and medical obligation under the right to health [18], which turns it into a mandatory competence for clinicians and nurses caring for patients with cancer. However, optimal pain management remains a challenge.

This article aims to briefly overview current knowledge on cancer-related pain assessment and treatment, and update on new approaches trends. We will also provide some insight on the lower- and middle-income countries (LMICs) context and highlight barriers and opportunities to reduce the existing gap between scientific evidence and pain management strategies implementation and delivery.

## Cancer-related pain

### Epidemiology

According to WHO estimations, there is an increasing prevalence of cancer worldwide, with over 18 million new cases and 10 million deaths in 2020 [19–21]. Although its incidence in LMIC is generally lower than in high-income countries (HICs), patients in LMIC frequently present with advanced disease leading to higher rates of morbidity and mortality [22]. Furthermore, pain management is more often inadequate on account of both limited resources and the low hierarchisation of the problem [23].

Within 2018 and 2019, a survey was conducted among professionals in LMIC and HIC, regarding knowledge, beliefs and barriers in pain management. It is worth mentioning that more than 80% of the respondents in HICs were satisfied with achievements in terms of pain therapy, against 59% in low-income countries [16].

Cancer-related pain remains the most common and burdensome consequence of the disease and its treatment [24]. A systematic review and meta-analysis published in 2016, shows that approximately 55% of those undergoing active treatment experienced pain, while the prevalence was over 64% in people with advanced disease. More than a third reported persistent pain after completing curative treatment, and 38% of all patients described moderate to severe pain (numerical rating scale score >5) with an evident negative effect on HRQL [1]. A pan-European survey of cancer patients also confirmed pain multidimensional impact and interference with daily life activities in almost 70%, often sub-estimated by professionals [5].

A more recent update on pain prevalence in patients with cancer shows a slight decline in both global occurrence and severity of cancer pain, compared to previous data. The overall prevalence of pain was 44.5%. Moderate to severe pain was experienced by 30.6% of the patients, a lower proportion in comparison with prior investigations. Nevertheless, pain prevalence remains high (particularly in treatment groups in which pain rates were significantly higher compared to those reported by cancer survivors) [25]. Conspicuously, studies from South America, Asia and Africa showed higher pain rates against studies from Europe (*p* = 0.033, *p* = 0.016 and *p* = 0.000, respectively). Moreover, studies from Africa described a considerably higher prevalence of pain compared to studies from all other continents [24].

Figures show that despite scientific advances over the last decades, pain in cancer patients is still a widespread problem [26]. The cancer burden is steadily increasing and so is cancer-related pain, which highlights the need for serious attention to pain relief as a global health care priority.

### Keystones in pain assessment

A comprehensive assessment is the first and essential step to pain control. It should start with a careful clinical interview with the patient and/or family, gathering information about the patient’s medical history, pain characteristics and psycho-social circumstances that may contribute to the symptom experience. Together with a thorough physical examination and diagnostic tests (when necessary), it will provide professionals with relevant information about pain quality and impact, as well as potential underlying mechanisms, causes and modulating factors. Consideration of these data should orient a more accurate and effective therapeutic approach.

- Types of pain

Pain in cancer patients is usually caused by the direct effect of cancer (*cancer-related pain*), cancer treatments (c*ancer-therapy related*) or by different causes other than cancer or its treatments (*unrelated painful conditions*) [27–29].

It can be sorted according to mechanism [30–34], in (Table 1):

1. *Nociceptive pain*, due to *somatic or visceral* involvement- such as soft tissue infiltration or distention of viscera,
2. *Neuropathic pain *– secondary to nerve compression or injury,
3. *Mixed* pain. Cancer pain is often of mixed etiology [35]. Bone metastases, for example, result of inflammation and remodeling and nervous system damage by the tumor [1, 36–38].

20% to 40% of patients with pain present a neuropathic component linked to higher intensity and impact, and poorer intervention outcomes [39].

Pain may change over time: It can be* continuous* or *episodic*. Identifying its pattern is paramount for the appropriate timing of pain treatment [40]. The presence of ongoing pain requires the prescription of fixed dose ‘the clock’ analgesia. On the other hand,* breakthrough pain* may need ‘rescue’ or additional dosing.

Breakthrough pain describes sudden transitory exacerbations of pain that may occur predictably (e.g., associated with a specific event or movement) or unpredictably (e.g., colic, stabbing pain associated with nerve injury) [41–43]. It is usually intense and short, rarely longer than an hour. It is common among cancer patients, and it often appears on a background of well-controlled baseline pain.

Cancer pain syndromes can be also classified into *acute* or *chronic*. Although traditionally, chronic pain is considered as pain that lasts longer than 3 months [44], it is not only a matter of time. Acute and chronic pains are distinct entities.

Acute pain presents suddenly and is shorter. It is usually intense and accounts for an underlying injury, disease or threat. It generally lasts from some minutes to less than 3 months and disappears when the cause is solved or healed. It is frequently accompanied by a ‘fight or flight’ response, also seen in acute anxiety. Acute cancer pain may be provoked by medical procedures or acute complications within the course of the disease, such as mucositis, acute intratumoral bleeding, visceral acute obstruction or bone fracture [45].

Chronic pain is more common, and it is generally cancer or treatment related [46–48]. Pain secondary to musculo-skeletal involvement or neural compression by the tumor is frequent. Sometimes, it appears as an adverse effect of surgery, chemotherapy or radiation. Unrelated conditions should also be considered [5, 49]. In chronic pain, vegetative features such as weight loss, insomnia, fatigue, inattention and loss of appetite, are prevalent. These symptoms overlap with those of depression, to which it is strongly related. Differential diagnosis is challenging. Even more, they frequently coexist, leading to a worse HRQL and poorer treatment outcomes [50, 51].

Chronic cancer pain experience results from physical and psychosocial factors that define the patient´s subjective perception. It is associated with negative or catastrophising beliefs, usually related to pessimism, ideas of disease progression and even death [52–54]. Persistent untreated pain would inevitably impact patients’ behavior and coping strategies. Mechanisms of plasticity may be also involved and should be acknowledged.

- Pain characteristics

Pain assessment should be integral, individualised and regular. Self-report is always the gold standard and professionals should encourage patients to talk about their pain when they are able to express themselves verbally [55]. As previously stated, pain quality descriptors suggest underlying mechanisms and will orient a more accurate approach [56]. For nonverbal patients (moderate or severe cognitive impaired, intubated or unconscious), reports should be taken from family or caregivers together with the identification of potential causes of pain and behavioral observation [57].

Mnemonics may be useful to ensure every aspect of pain is considered, including quality, intensity, location, onset, temporality, radiation and triggers. Widely spread mnemonics are: OPQRST, SOCRATES (Table 2), OLDCARTES, COLDSPA, WILDA and so on. [33, 58, 59].

Pain description through self or surrogate report is essential, but only the initial part of the evaluation. Patients may present more than one symptom, pain in multiple sites or even referred pain. Complete assessment implies the ability to pinpoint patients´ needs and make a diagnosis. This demands communication skills as well as grasping the relevance of general and neuroanatomy, and nociception, considering behavioral indicators and identifying potentially painful pathological processes. Pain scales or behavioral assessment tools are useful in practice.

Understanding pain intensity and impact, together with underlying mechanisms and causes, allows professionals to choose the most appropriate therapeutic approach and anticipate potential responses to specific interventions [60]. Medical history, clinical examination and complementary tests, when necessary, should lead to better pain syndrome definition and results.

- Pain assessment tools

Pain assessment is critical for a comprehensive therapeutic approach. Therefore, the use of systematic assessment practices is recommended [61]. Given the subjective nature of pain, uni or multidimensional pain measures could help to quantify the symptom, evaluate it more accurately, facilitate team monitoring and assess the effectiveness of interventions [62]. Pain assessment tools provide more objective information about the symptoms both in patients who communicate verbally or those without verbal communication ability [63]. Considering variability between scales, Caraceni *et al* [64] recommended the use of standardised pain assessment scales in clinical and research palliative care.

**Figure 1.**
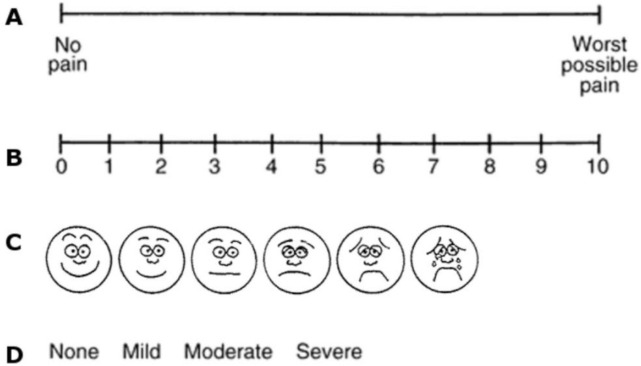
Unidimensional pain scales. (a): VAS, (b): NRS, (c): Wong–Baker faces pain rating scale, (d): Verbal rating scale (VRS).

Pain intensity is widespread measured with a numeric scale (NRS), a unidimensional eleven-point scale in which 0 stands for ‘no pain’ and 10 represents ‘the worst possible pain’. The NRS and the Visual Analogue Scale (VAS) are reliable tools, validated in different languages and settings [65]. Other categorical or pictorial scales are also commonly used [66] (Figure 1).

Furthermore, multidimensional tools may gather additional information, including other characteristics of pain, level of interference with daily living and affective aspects [67]. The McGill Pain Questionnaire and the brief pain inventory BPI are two of the most recommended multidimensional tools [68, 69].

Specific questionnaires for the assessment of neuropathic pain (DN4, *Neuropathic Pain Questionnaire*, *Neuropathic Pain Scale*) [70, 71] and cancer pain in nonverbal patients are available [57, 63, 72, 73]. In any case, the selected instrument should be reliable and valid for one particular population and setting, and at the same time, easy to administer and clinically useful [57, 74, 75]. The most used and validated scales for non-verbal patients in Palliative care are the Behavioral Pain Scale, Checklist of Nonverbal Pain Indicators, Multidimensional Observational Pain Assessment Tool and other observational pain behavior rating scales for specific populations (e.g., PAINAD and DOLOPLUS2 in Dementia) [63, 76–82].

Despite proven benefits, scales should never replace clinical judgment based on an open dialogue with patients and families and a deep clinical examination. As a complex experience, pain includes physical, emotional, cognitive, behavioral and social dimensions which contribute to the overall perception. All these aspects should be carefully evaluated.

- Psychosocial aspects of pain

Forty years ago, R. Twycross affirmed: *‘pain is more than a sensation. It is a dual phenomenon. One part is the perception of the sensation and the other the patient’s psychological reaction to it. It follows that a person’s pain threshold will vary according to mood, morale and meaning. For any given noxious stimulus, the pain experienced varies from ache to agony and depends on the psychological reaction of the sufferer to his discomfort… Much can be done to alleviate pain by explaining the mechanism underlying the pain (this reduces anxiety) and by a continuing concern for the patient (this raises morale). Ignoring mental and social factors may result in other- wise relievable pain remaining intractable’* [32, 53]. Pain perception and expression depends not only on noxious stimuli or physical damage, but also on the patient’s personality, previous painful experiences, pain understanding and interpretation, individual and cultural beliefs and attitudes towards the symptom and the underlying disease [83–86].

Unrelieved pain generates emotional symptoms and reactions, such as anxiety, depression, fear, anger and frustration, which in return, increase pain perception [51, 87, 88]. Moreover, evidence shows that pain intensity is consistently higher in patients with anxiety or psycho-spiritual distress, factors that contribute to more difficult management and an increased risk of inappropriate use of pain medication [59, 89, 90].

As Twycross has pointed out, the lack of positive meaning and uncertainty intensifies pain severity; on the contrary, life satisfaction, realistic hope and expectancies and an empathic patient-doctor relationship may reduce pain intensity with a minor need for analgesics and sedatives [32, 45, 53] (Table 3). Recently published research evaluated simultaneous brain activity (fMRI hyper-scanning) from chronic pain patients and clinicians during patients’ reception of evoked pain. Brain activation was assessed in patients with and without patient-clinician interaction. Results showed that clinicians’ support increased patients’ activation in prefrontal and somatosensory areas, as well as concordant activation in both patient and clinicians’ brains. At the same time, interaction seemed to reduce pain severity and enhance (self-reported) therapeutic alliance in comparison with patients stimulated in isolation. This study infers mechanisms that underlie the social modulation of pain and demonstrates the influence of empathic care in pain management [91].

Ten to twenty percent of the patients present difficult or refractory pain (pain that does not respond to standard therapy). Evidence reveals that psychological factors together with neuropathic involvement and breakthrough or episodic pain are some important negative predictive indicators in pain management [92]. In those cases, comprehensive re-assessment is compulsory and associated emotional, existential and social factors should be considered and intervened [93]. Cicely Saunders ‘total pain’ confirms the multidimensional nature and impact of cancer pain and the need for a holistic and multidisciplinary approach [94].

**Figure 2.**
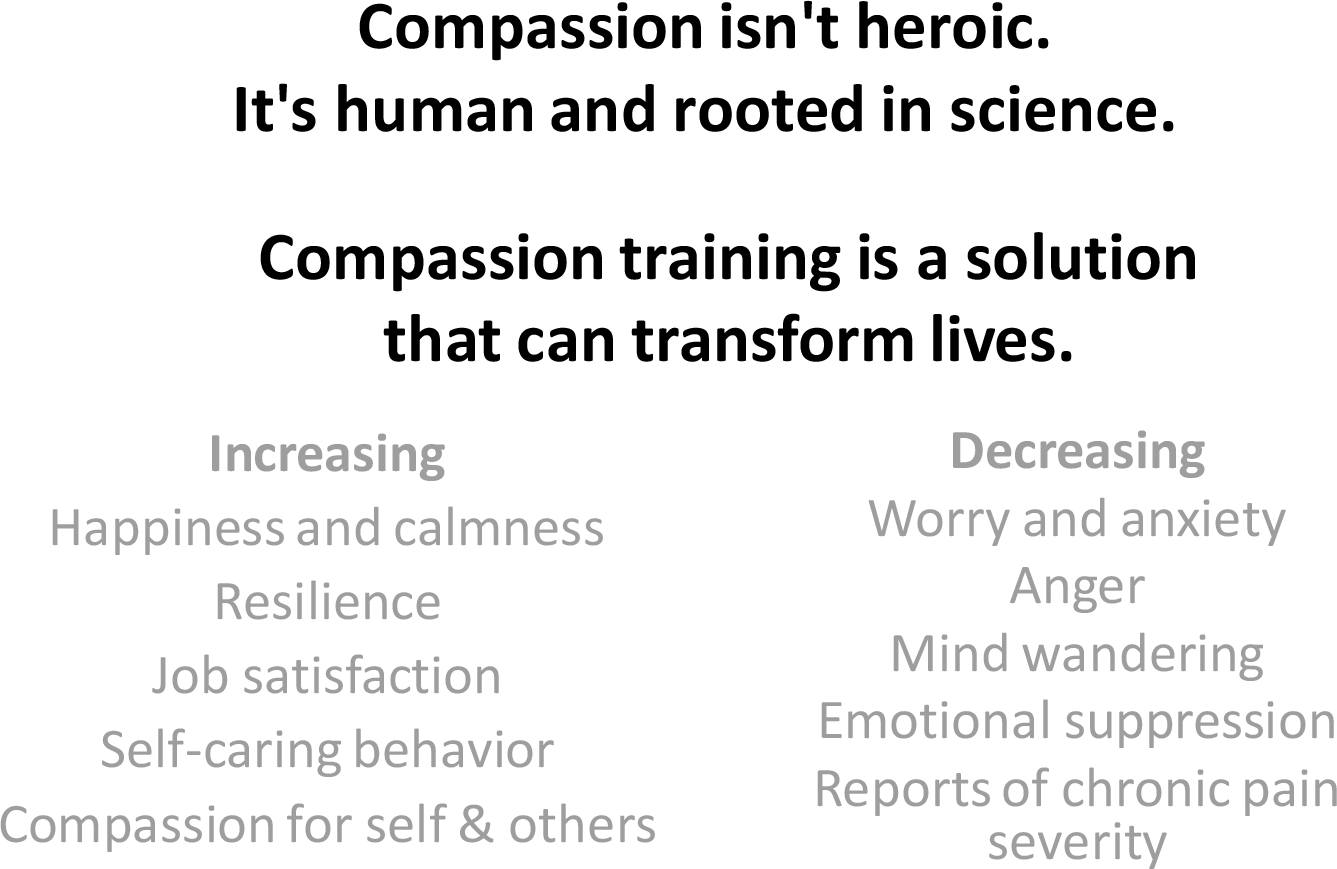
Inspired by: https://www.compassioninstitute.com/about/why-compassion-training/.

Within the last decades, there has been a growing interest in the importance of compassionate care in health matters [95]. Compassion is defined as *sensitivity to suffering, the ability to understand another person’s feelings, combined with a willingness and motivation help to relief or prevent that suffering*. It is an essential aspect of high-quality healthcare, particularly in palliative care settings [96–98] (Figure 2). In line with this, Brito *et al* [99] affirm that Compassion Cultivation Training Programs may enhance professionals’ self-compassion and compassion for others with beneficial consequences on therapeutic outcomes and patients’ quality of life. In addition, there is growing evidence that novel interventions that blend individualised care, mind–body–spirit approaches, together with pharmacological plans are more successful than usual care strategies in helping patients on opioid discontinuation or tapering [100].

## Management of cancer pain

Cancer pain management aims to provide a multidimensional proactive therapeutic approach to prevent and relief pain and suffering, searching for the best possible quality of life and performance in daily living. Traditionally, a two-point reduction on the NRS or a 30% decrease of baseline pain would represent a successful outcome in pain management [101, 102]. However, besides minimising pain intensity, focus should also be set on improving patients’ functionality, and reducing emotional, social and existential impact of the symptom on their lives and those of their beloved ones [103, 104].

Agreement about goals of care between patient and team is central within a patient-centered strategy, particularly when talking about cancer pain [105]. Goal setting should be an individualised process in which the patient would define goals in terms of comfort (acceptable or tolerable level of pain) according to his own perspective. Patients’ participation at this point is crucial, and professionals’ aim would be to facilitate realistic expectations as well as to help the patient to achieve proper relief [106]. Close follow up is required in order to check on the effectiveness of interventions, need for dose titration and side effects management [59].

A comprehensive approach refers to **cause-specific treatments** when possible and appropriate symptomatic **nonpharmacological** and **pharmacological** interventions, delivered by an interdisciplinary team [22, 107, 108]. This strategy defines integrative pain care, combining different modalities in a collaborative framework [22]. Mainstay aspects of pain control are pointed out and updated. Cause-directed treatments are beyond the scope of this review.

- Nonpharmacological strategies

Nonpharmacological interventions have shown to reduce cancer pain intensity and increase patients’ HRQL through different mechanisms and techniques [109]. They include different practices, therapies or modalities that can be divided in subcategories: physical therapy (involving exercise, acupuncture, massage, thermotherapy – cold and heat – and transcutaneous electric nerve stimulation), psychosocial therapy (e.g., cognitive and behavioral therapy), nutritional advice and other complementary or alternative therapies [110]. These strategies are usually applied in combination with pharmacological or interventional treatments.

In addition, some of them improve psychosocial functioning and integral wellbeing, integrating mind, body and spirit [110, 111]. Complementary integrative therapies (CIT) may relief a specific type of pain or help with associated anxiety, fatigue or negative mood, although long-lasting effect is not clear. Few randomised controlled trials (RCTs) support the analgesic efficacy of hypnosis, acupuncture and music therapy. However, most benefits are based on clinical observation, or professional and clients’ perspectives. Still, their use is grossly underestimated [110, 112–114].

In general, CIT are associated with less treatment burden and fewer adverse effects. Further high-quality studies are needed. In spite of this, current evidence provides promising data about the role of CITs such as mindfulness, cognitive therapies and acupuncture in pain relief, quality of life and safety within palliative care settings [113–115]. Moreover, they are believed to enhance the sense of control over pain and coping resources [116].

Non pharmacologic interventions should be a part of a multimodal and interdisciplinary approach, in which social and spiritual needs are systematically assessed and taken care of [117].

- Update on pharmacological pain treatment

For decades the empirical WHO 3 step analgesic ladder has remained the keystone to cancer pain management education worldwide. Recent emerging knowledge from specific pieces of research, and/or systematic reviews supports new recommendations:

### Step 1: NSAIDs and paracetamol (acetaminophen)

NSAIDs and paracetamol constitute the first step of the WHO analgesic ladder and have been added as important components of the second and even third step [118]. Their role is still endorsed by several international guidelines for mild pain, as single drugs, or in moderate and severe pain in combination with opioids [118, 119]. Recommendations also include the addition of NSAIDs, mainly when a clear inflammatory mechanism is involved – like in metastatic or primary cancer-induced pain [120]. Despite this, their use in cancer pain management remains controversial [121]. There is a paucity of data from the past 20 years regarding their efficacy in cancer pain management or underpinning benefits when adding an NSAID to second or third-step opioids [15]. A systematic review published by Magee *et al* [122] suggests that there is a lack of high-quality evidence regarding the analgesic efficacy of NSAIDs in cancer pain, with short-term studies and heterogeneity in outcome measures, limiting the ability to draw meaningful conclusions. Furthermore, evidence for additional analgesic efficacy as adjuncts or an opioid-sparing effect is inconclusive [120, 123].

Though evidence for NSAIDs prescription in cancer pain is not well supported, they should be taken into account on an individual basis, considering potential benefits, as well as potential vascular, gastrointestinal and renal adverse effects [121, 122]. The use of proton pump inhibitors has been shown to reduce the risk of gastrointestinal toxicity. However, the consequences of long-term prescription of these drugs must be considered [124].

Quality evidence for or against the use of paracetamol (or acetaminophen) in cancer-induced pain is lacking [123, 125]. Nevertheless, its prescription as a first-line medication in mild pain, or in combination with opioids is still widely utilised [37, 125]. In fact, numerous formulations incorporate acetaminophen with an opioid within a single tablet. A recent Cochrane systematic review in 2017 concluded that adding paracetamol to a daily regimen of 60 mg or more of oral morphine results in no additional benefit in terms of pain relief, quality of life or patient satisfaction or preference [125]. Moreover, risks for hepatotoxicity and treatment burden were mentioned [126–128]. Four RCTs compared high doses of morphine with and without acetaminophen, and the effects of both groups were similar. Based on these findings, acetaminophen may not be of benefit when used in combination with third-step opioids [15, 126, 129].

### Steps 2 and 3: Opioids

Opioids should be offered to patients with moderate-to-severe pain related to cancer or active cancer treatment unless contraindicated. They should be initiated as immediate release at the lowest possible dose to achieve acceptable analgesia and patient goals, with early assessment and frequent titration. For patients with substance use disorder, clinicians should work in collaboration with palliative care, pain and/or substance use disorder specialists to determine the optimal approach to pain management. Opioid adverse effects should be monitored. Sustained strategies for prevention and management should be implemented [15, 130].

Initiation of opioid therapy is a delicate and challenging process. The distinction between so-called weak opioids (second step) and strong opioids (third step) is controversial, as strong opioids can be used at low doses to replace weak opioids [131]. Fallon *et al* [132] provided some evidence that a two-step approach appeared to be at least as effective and safe as a three-step ladder strategy, and probably a less expensive option. RCT confirmed that an initial low dose of morphine or other strong opioid may achieve earlier and more significant relief in comparison with weak opioids. Despite this, second-step opioids may be still necessary and useful due to hindrances in strong opioids availability, accessibility and acceptance [132].

There are different clinical situations that may require different approaches for patients with moderate-severe pain [133]. Of interest, the analgesic benefits conferred by opioids must be carefully balanced against the development of adverse effects, as they could negatively affect treatment adherence and HRQL. Many adverse effects can be anticipated and mitigated by the appropriate use of symptomatic drugs [134]. The key determinants of adverse effects related to opioid therapy include both patient-related and medication-related factors. Concurrent use of some drugs exacerbates side effects. For example, the concomitant use of benzodiazepines could significantly worsen the patient’s cognitive function or risk for respiratory depression. Strategies to manage adverse effects are based on opioid dose reduction, opioid switching or symptomatic treatment [135].

Codeine and tramadol are commonly prescribed with good efficacy for moderate pain. In seven out of eight RCT studies, codeine was significantly more effective than placebo, with a poorer response related to morphine and oxycodone. However, its use is still widespread, considering its low price and easier access in most countries. On the evidence available, it is conditionally recommended for moderate pain [129]. It is considered a pro-drug undergoing CYP2D6 metabolism, with concerns regarding genetic abnormalities impacting metabolism [136, 137].

Regarding tramadol, most RCT studies reported similar analgesic efficacy and safety compared with other weak opioids and low doses of strong opioids. Recommendations support its use for moderate cancer pain, when administration of strong opioids is not possible or due to patient preferences or medical judgment with a moderate level of evidence [129].

Data on the use of buprenorphine for cancer pain is limited [136]. Analgesic potency is comparable to strong opioids, although there is still not consensus about conversion ratios and opioids equipotency [129]. Its profile as a partial agonist showed proper pain response with benefits in terms of adverse effects. Moreover, it has proven to be a good alternative for moderate to severe pain in the elderly population and renal impairment [129, 138, 139]. Several RCTs show at least equivalent pain relief in comparison with traditional third-step opioids, with similar or even better tolerance and ceiling effect for respiratory depression [139–141]. However, differences regarding routes of administration, clinical experience, formulations availability and costs across countries should be taken into account. Sublingual (SL) and transdermal (TD) routes achieve similar bioavailability rates. TD buprenorphine achieves a slower concentration increase and no peak effect in comparison with SL, which makes the latter more suitable for rescue analgesia [142]. Intramuscular buprenorphine is not recommended for cancer pain management.

As mentioned above, prescribing strong opioids, such as morphine, methadone or fentanyl, for moderate pain in opioid-naïve patients appears to be effective and safe. Suggested initial doses of 15–30 mg/d of oral morphine or 6 mg/d of methadone showed to be appropriate, with a minimum requirement of titration within the first month [143].

Morphine has been traditionally considered as the drug of choice for moderate to severe pain (level of evidence: high) [144]. According to a Cochrane systematic review, patients receiving morphine analgesia achieved adequate relief in more than 90% of the cases. It is often the first option according to its effectiveness, wide availability, predictable pharmacokinetics, cost and experience. Reported common adverse effects were constipation, drowsiness, nausea, dry mouth and vomiting [129, 144]. An increasing number of RCT that compared methadone, hydromorphone, oxycodone or fentanyl patches with other opioids, showed similar analgesic and adverse effects. Few studies reported lower rates of constipation or vomiting with fentanyl against morphine. Other differences between opioids have been reported, although evidence is not strong enough to favor one over the others [129, 145].

Methadone has unique properties that make it a valuable option in certain scenarios, such as neuropathic pain (due to its NMDA receptor antagonism) or renal impairment (since it does not have active metabolites). It can provide more effective pain relief when converting from another opioid, in patients with insufficient analgesia, high tolerance development or unacceptable adverse effects. Besides potential advantages, it has some specific considerations and risks. Due to its long half-life and variable pharmacokinetics, dosing and titration may be complex. Additionally, conditions for methadone cardiac side effects appearance (QT prolongation and increased risk of arrhythmias) and drug interactions are not fully determined. There is still a lack of experience in the administration of methadone in most countries. Consequently, its use is sometimes limited to opioid switching for refractory pain with poor response or intolerance to prior opioids. This strategy has shown to be effective in terms of analgesia, safe and cost [129, 145, 146]. However, recent retrospective studies and case series demonstrated safety and efficacy of methadone as a first-line opioid for cancer pain management, suggesting that initial low-doses of the opioid and rescue doses provide rapid relief with minimal need for titration and adverse effects [143, 147, 148]. In any case, careful dosing and monitoring are required.

According to current evidence, the clinical decision on opioid selection should be based on patients’ history, type of pain and other factors such as professional experience, pharmacokinetic properties, toxicity profile, cost and availability of each drug. Data remains insufficient to recommend for or against the use of genetic testing, such as for polymorphism of CYP2D6, to guide opioid dosing.

For its part, titration should be tailored to individual experience and response, bearing in mind the patient’s previous exposure to opioids, potential interactions, organ impairment or comorbidities [15]. Oral immediate-release formulations should be of choice for initial treatment, providing rapid onset of relief and flexible titration and management of breakthrough pain. Although current titration protocols suggest dose adjustments by increasing 25% to 50% (up to 100%) of the previous daily dose, pain intensity and patient conditions such as frailty and organ function should be evaluated and considered [15, 130].

Intravenous (iv) analgesia may be required in several situations where effective and rapid pain control is essential. Parenteral opioid therapy may allow for accurate and fast titration, with almost immediate relief. As soon as the patient’s condition has stabilised and if oral medication is well tolerated, a transition to oral opioids may be necessary and appropriate. Conversion charts would provide the relative potency of different opioids and routes of administration [149].

### Adjuvants

Adjuvants are usually prescribed in combination with opioids, in order to either enhance analgesia (co-analgesics) or improve opioids side effects. Antidepressants and anticonvulsants are widely used as co-analgesics, particularly in neuropathic pain. However, quality evidence of their support in cancer pain is scarce.

Antidepressants. Six out of nine RCTs reported the effectiveness of antidepressants such as duloxetine, venlafaxine, amitriptyline, imipramine and fluvoxamine in cancer. Benefits of this group in neuropathic or metastatic bone pain treatment have been reported [29]. However, it is important to note that the incidence of adverse effects with antidepressants was higher compared to a placebo. Cardiovascular and anticholinergic side effects are often limiting [33]. Despite this, the combination of antidepressants with opioids is conditionally recommended based on individual opioid response and tolerance levels [129].

Anticonvulsants. Six out of seven studies of gabapentinoid or gabapentinoids in combination with opioids demonstrated effectiveness. Both gabapentin and pregabalin are widely used for neuropathic pain of various etiologies. Additional benefits were reported in terms of anxiety and insomnia In one double-blind RCT in neuropathic cancer patients, pregabalin has been shown to provide better relief and functional status against gabapentin and amitryptiline [33, 129].

According to data, the occurrence of negative side effects from anticonvulsants or gabapentinoids was higher compared to a placebo. Therefore, it is conditionally recommended to use these medications in combination with opioids for metastatic bone pain or neuropathic cancer pain as additional pain relief, with a low level of supporting evidence [129].

Both antidepressants and anticonvulsants present different mechanisms of action. Therefore, they can be combined when one single drug is not effective. Research has shown that duloxetine added to opioid-pregabalin therapy provided additional clinical benefit in alleviating neuropathic cancer pain [29, 150].

Antiarrhythmics. A limited and small RCT provides sparse evidence for the use of subcutaneous lidocaine for neuropathic cancer pain in combination with opioids. Potential benefits and adverse effects should be considered individually within other possible interventions [129].

Ketamine. Although oral or parental ketamine has been used for chronic neuropathic pain, there is a lack of quality evidence for its recommendation in cancer patients [29, 129]. According to its NMDA receptor antagonism, it may be an effective adjuvant against neuropathic cancer pain related to central sensitisation, by reducing opioid requirement and risk of tolerance. Nonetheless, its mechanism is not fully understood [29]. Additionally, neurological and cardiovascular side effects limit its use in critically ill patients. For these reasons, some guidelines mention it as a third-line analgesic, requiring a controlled setting for administration and expert training [129, 135].

Steroids. Steroids have been found to be effective in relieving cancer pain when used in combination with other treatments (opioids, other adjuvants or interventional treatments), particularly for inflammatory, visceral and neuropathic pain [45, 151]. However, there is limited well-designed large-scale RCT evaluating their efficacy and safety. Besides most existing studies focus on short-term outcomes and different populations and dosages. Their mechanisms of action may be related to anti-inflammatory effects and potential neuroinmunological modulation [151]. Side effects are frequently described, particularly in the geriatric population and long-term use, although they are not fully evaluated or compared against controls [82, 129, 151, 152]. According to available data, the recommendation for their use is limited to cancer pain associated with nerve compression, transitory pain flares following radiotherapy, and headaches associated with intracranial hypertension [128]. In practice, steroids are widespread prescribed as adjuvants in cancer pain, generally, with significant but temporarily relief.

- Interventional therapies

Interventional procedures constitute a good alternative for uncontrolled pain or systemic analgesia with intolerable side effects [153]. Techniques such as sympathetic blocks, neuraxial analgesia or peripheral nerve blocks are recommended in specific pain syndromes showing rapid and effective relief and the possibility of reducing systemic pharmacological therapeutics [154] (Table 4). Recent controlled trials support the benefit of invasive procedures for visceral abdominal pain, showing significant analgesia and increased quality of life [155, 156]. For example, celiac plexus block provides good relief in pancreatic cancer pain, although proper timing is still questionable [156, 157].

Evidence for the use of invasive procedures in cancer pain is limited yet [158, 159]. However, their indication should be timely considered, together with other analgesic strategies, rather than reserved as a last option [158]. Early use of these interventions alongside or before opioid therapy can reduce symptoms burden, and opioid requirement, and potentially impact survival [160]. Hochberg *et al* [159, 160] emphasise the importance of integrating interventions at earlier stages, referring to ‘the handrail’ of the WHO ladder when possible and appropriate. Potential benefits, feasibility and safety should be assessed in each case, according to pain characteristics, patient’s state and life expectancy and other available alternatives [154]. Procedural interventions require professional skills, proper equipment, and an accurate indication, based on a comprehensive and interdisciplinary assessment [159].

Intraspinal analgesia refers to the administration of drugs directly into the epidural or intrathecal spaces. It can provide targeted effective pain relief with fewer systemic side effects in comparison with oral or intravenous medication. Opioids like morphine, hydromorphone or fentanyl have been traditionally delivered alone or in combination with other adjuvant medications. Local anesthetics such as bupivacaine and lidocaine are commonly used as well. Novel drugs such as sufentanil or ziconotide (a selective N-type calcium channel blocker) may offer potential advantages in terms of efficacy and safety, although specialised training and equipment are required, considering professionals’ expertise, resources and potential severe side effects [137].

### Novel therapeutics

Although opioids are well-established in managing cancer pain, some patients show limited response to opioids due to genetic factors or particular pain profiles [149]. Additionally, concerns over side effects and addiction often lead to prescription delays or inadequate dosing. Novel therapeutics continue to emerge, offering alternatives to traditional treatments. Some of them include: opioid analogs; cannabinoids; monoclonal antibodies; nerve growth factor inhibitors; transient receptor potential vanilloid 1 antagonists and other targeted therapies. Furthermore, new non-pharmacological interventions are being explored.

Tapentadol is a novel analgesic that binds to the mu opioid receptor and inhibits the reuptake of noradrenaline. Its dual mode of action may explain an opioid-sparing effect and promise positive outcomes in the management of arious types of pain, including nociceptive and neuropathic pain. Unlike tramadol, tapentadol is a more potent mu agonist and does not need liver activation. According to clinical trials, it shows significant relief of moderate to severe pain, and less gastrointestinal side effects than traditional strong opioids. However, evidence, particularly in the context of cancer, is still scarce [29, 137, 161]. Further studies and clinical trials are needed to establish its efficacy and safety profile and to better inform treatment decisions [162].

Cannabinoids, such as THC and CBD found in the cannabis plant, have shown potential therapeutic effects including pain modulation, effects on mood and appetite regulation in cancer patients. While some studies have shown promise in alleviating cancer pain in patients who do not find relief from opioids, overall evidence is inconclusive. Evidence suggests that they may have a role in alleviating hyperalgesia and allodynia, particularly in neuropathic pain. However, recent RCTs have not consistently demonstrated significant analgesic advantages of cannabinoids over placebo [163, 164]. Adverse effects associated with cannabinoids include dizziness, dry mouth, nausea, confusion and somnolence. These side effects may limit the broad usage of cannabis-based medicines for cancer pain management [165–167]. Further well-designed RCTs with larger sample sizes are needed to clarify the optimal dosage and effectiveness of cannabis-based therapies in treating cancer-related pain.

Botulinum Toxin Type A (BoNT-A) is a neurotoxin produced by *Clostridium botulinum* that inhibits the presynaptic release of acetylcholine in muscle tissue. Studies have shown that local injections of botulinum neurotoxins may help alleviate nociceptive and neuropathic pain related to cancer or its treatments. There is no consensus regarding the appropriate dosage of BoNT-A, which would vary depending on the characteristics of pain, the type of toxin and individual response [168, 169].

Denosumab is a monoclonal antibody and targeted RANKL inhibitor. It has emerged as a valuable treatment option for patients with bone metastases. Studies have shown denosumab to be more effective than zoledronate in delaying the return of pain in breast and prostate cancer [170, 171]. Therefore, it is considered an alternative to bisphosphonates for managing bone pain and delaying the progression of skeletal-related events in patients with metastatic cancer from solid tumours or myeloma [37]. It offers advantages such as subcutaneous self-administration, no need for hospitalisation or dose adjustment in renal failure [172]. Preventive dental measures are recommended before starting denosumab to reduce the risk of osteonecrosis of the jaw [37].

Other new molecules for nociceptive or neuropathic cancer pain management are under current research, but further quality RCTs are still needed [173]. That is the case of oliceridine (opioid analog), growth factors inhibitors [137], new target toxins (tetradotoxin [174, 175], TRPM8 activator menthol [176] lemairamin and protease-activated receptor 2 antagonists [176–179].

### Management of opioid-induced adverse effects: emerging knowledge

- Constipation

Clinical evidence highlights that prevention of opioid-induced constipation (OIC) is essential. Both, dietary interventions and prophylactic use of laxatives at the start of opioid treatment are strongly recommended [129]. Non-pharmacological measures include hydration and nutrition, ensuring privacy during defecation, using a commode or footstool, and the availability of a caregiver. Abdominal massage may be of value.

Although these strategies are still mentioned in the context of OIC, their efficacy in treating the syndrome may be limited and pharmacological approach seem to be needed. Furthermore, increased dietary fiber may be discouraged in cancer patients, which may lead to bowel obstruction or abdominal discomfort. Practice guidelines usually recommend regular doses of (osmotic or stimulating) laxatives as first-line treatment for OIC, based on efficacy, convenience safety and cost [32, 129, 180].

Good quality evidence supports the use of peripherally acting mu-opioid receptor antagonists such as naldemedine, naloxone and methylnaltrexone, for preventing and managing OIC, in terms of efficacy and safety. However availability and cost may be limitations in practice. Therefore, they are recommended for OIC refractory to other treatments [129].

Emesis. Opioid-induced nausea and vomiting (OINV) appeared to be more frequent with tramadol and morphine related to other opioids [181]. No significant difference was reported in regard with different administration routes. The administration of antiemetics for OINV is recommended, although preventive prescription should be only considered in specific clinical situations [130]. First-line antiemetics for nausea and vomiting in advanced cancer are metoclopramide and haloperidol. Methotrimeprazine (also called levomepromazine) and olanzapine are considered as second-line medications. For patients reporting previous episodes of nausea during past exposure to opioids, prevention may include pretreatment with metoclopramide around the clock for the first few days of opioid therapy, with gradual weaning of the antiemetic [32].

Sedation. Conclusions from four RCTs on the treatment of opioid-related sedation demonstrated that methylphenidate improved drowsiness, cognitive function, motor function and mental activity. Adverse effects were comparable to placebo. On the other hand, caffeine showed no significant effect on opioid-induced sedation. The effects of pemoline are unclear. Most experts agree on the importance of reviewing potential drug-drug interactions that may be affecting opioid metabolism (new-onset sedation with stable opioid dosing is generally related to the addition of other sedating agents). Moreover, opioid dose reduction or switch should be evaluated. ASCO Guideline considers naloxone for patients receiving opioids with benzodiazepines, gabapentinoids or other sedating agents [130].

## Specific clinical pictures: recommended therapies and considerations

### Bone pain

All patients with unrelieved metastatic bone pain should be considered for external beam radiotherapy or radioisotope treatment. Single-dose fractionated radiotherapy should be used when radiotherapy is indicated and available. (Strong recommendation; high-quality evidence) [28].

In adults (including older persons) and adolescents with bone metastases, bisphosphonates should be evaluated in order to prevent and treat bone pain. (Strong recommendation; moderate-quality evidence) [28].

Other treatment modalities (radioisotopes, monoclonal antibodies) are sometimes administered for diffuse bone pain that cannot be treated with radiotherapy or fail to current management.

### Neuropathic pain

Up to one third of patients with cancer have neuropathic pain of some kind. When directly caused by cancer, nerve compression generally precedes nerve injury. Inflammation plays a role in the peripheral and central sensitisation that occurs: some pains are ‘mixed pains’ [30]. Strong opioids are used in moderate or severe cancer pain but also neuropathic pain caused by cancer or from other chronic nonmalignant conditions. First-line adjuvant choice for cancer-related neuropathic pain includes amitriptyline, duloxetine, gabapentin or pregabalin. They are also first-line choices for non-cancer neuropathic pain. Strong evidence about the best drugs to be used remains limited, as do therapeutic choices: because their efficacy and tolerability are comparable, choice is influenced by cost and individual circumstances, e.g., concurrent co-morbidity, lox mood and poor sleep. Treatment options for refractory neuropathic pain (non-respondent to opioids+/+antidepressants and gabapentinoid antiepileptics) should be managed only by specialists. These include, e.g., methadone, ketamine, oxcarbazepine, valproate and interventional analgesia. Their place, relative to each other, is uncertain and selection is influenced by local availability and expertise [32].

### Skeletal muscle pain

Unless associated with local inflammation (e.g., cancer invading muscle) skeletal muscle spasm does not respond to traditional analgesics, including strong opioids. All or part of a muscle may spasm in response to local pain, e.g., muscles adjacent to a fractured bone. A muscle cramp is a forceful and sustained muscle spasm. The mainstay of treatment for this condition is an explanation and non-drug therapies, e.g., local heat, massage and/or relaxation therapy. Some patients benefit from relaxant drugs (diazepam and baclofen) [32].

### Breakthrough pain

In patients receiving opioids around the clock, immediate-release opioids at a dose of 5%–20% of the daily regular morphine equivalent daily dose should be prescribed for breakthrough pain (ASCO Guideline - Type: Informal consensus, benefits outweigh harms; Strength of recommendation: Strong for prescribing immediate-release opioids for breakthrough pain, weak for dosing). Evidence remains insufficient to recommend a specific, short-acting opioid for breakthrough pain [130].

### Pain in patients with renal/hepatic failure

For patients with renal impairment currently treated with an opioid, clinicians may rotate to methadone, if not contraindicated. In this condition, the elimination of the drug and its metabolites is almost exclusively by fecal excretion, which makes it the most suitable option. Furthermore, dialysis removal is extremely low. Opioids primarily eliminated in urine, such as fentanyl, oxycodone and hydromorphone, should be carefully titrated and frequently monitored for risk or accumulation of the parent drug or active metabolites. Morphine, meperidine, codeine and tramadol should be avoided in this population, unless there are no alternatives. (ASCO Guideline - Type: Informal consensus, benefits outweigh harms; Strength of recommendation: Strong) [130]. If methadone is not available, fentanyl or buprenorphine are recommended. Parenteral, TD or other formulations should be considered. In case there are no other options, administration of the used opioid should be for only a short period with a low dose [129]. NSAIDs should be prescribed with caution.

There are no evidence-based guidelines for pain management in patients with liver failure. Opioids use remains challenging, on the bases of increased toxicity. OIC, sedative effects and sudden encephalopathy are common. In cirrhosis, metabolic pathways (oxidation, glucuronidation, first-pass metabolism) and protein binding are affected. In consequence, these drugs clearance is reduced and/or bioavailability increased, with increased half-life and peak concentrations [182, 183]. Methadone and fentanyl toxicity may not be increased due to reduced metabolism, although reduced dosing is required particularly in hypoalbuminemia, considering changes in protein bound. Low doses of intravenous fentanyl or oral or intravenous hydromorphone are appropriate options [184]. Reduced liver conversion of pro-drugs such as codeine via CYP2d6 explains the decrease or lack of analgesia with these opioids. For this reason, codeine, hydrocodone and oxycodone should be avoided [183]. In the same way, meperidine is discouraged due to the higher bioavailability and prolonged half-life of its toxic metabolite. In conclusion, opioids should be prescribed with caution. Lower doses and longer intervals are recommended. In all cases, close monitoring is required and discontinuation should be considered in case of inacceptable side effects.

Acetominophen overdose is a common cause of liver failure. However, it is still an adequate choice for mild or moderate pain, even in patients with liver dysfunction. Dose reduction from 4 to 2 or 3g/d is recommended for long-term use [185, 186]. NSAIDs should be avoided due to the increased risk of acute renal failure and varices bleeding in these patients [185–188].

### Barriers to pain control

Several factors have been pointed out as underlying reasons for suboptimal pain control. Experts from The Netherlands [189] described problems related to pain complexity; underestimation of pain; lack of specific professional knowledge; time constraints, as well as cultural perceptions or misconceptions about pain treatments [190, 191]. In addition, studies from LMICs reported [17, 192–194]:

- Limited availability and accessibility of opioids [3];
- Restrictive government policies and opioid regulation;
- Lack of resources and poor insurance coverage;
- Lack of education and training in pain assessment and management;
- Scarce pain specialists and centers;
- Lack of patient-doctors communication;
- Failure or delay in patients’ referral to specialists.

Concerns about opioid abuse or addiction, particularly in some countries, have influenced patients’, professionals’ and policy makers’ attitudes toward its prescription, even for cancer-induced pain. Patient-related barriers include less expression of pain, inadequate adherence to analgesics and fear for side effects or addiction [193–196]. For their part, strict opioid regulations, misconception and lack of specific training, hamper opioids availability and accessibility and entail undertreatment and low opioid consumption. It must be noted that Latin America’s situation about misuse is (up to date) different from the US or other countries. Opioid addiction rates and consequent mortality seem to be lower, especially in palliative care settings [196]. However, undertreatment is still a worrisome problem. In 2019, local experts gathered together to write a position paper that is expected to prevent opioid abuse and diversion, and improve pain management in the region [196]. Recommendations include: Constructing local statistics that would allow appropriate health policies development; fostering pain education programs (both at undergraduate and graduate levels); ensuring interdisciplinary approach and high-quality patient-doctor communication and promoting patient education and continuous support [193, 196–198].

## Final key recommendations

1. Systematic screening and comprehensive evaluation of pain are essential for individualised and effective cancer pain treatment, considering heterogeneity of pain and patients subjectivity [50, 51, 61, 199].
2. An integral and integrated approach that assures empathic communication and considers not only physical, but also, emotional, spiritual, social and economic variables is also crucial [200].
3. Non pharmacological interventions and impeccable pharmacological management, including appropriate prescription of analgesics, are the key in terms of efficacy and safety. Opioids are still the cornerstone of cancer pain relief, most times in conjunction with non-opioids analgesics and adjuvants [201]. Recommendations and guidelines are valuable tools in practice, but they should promote healthcare providers’ reflexive judgment in order to support appropriate decision making for each patient in his particular and unique context.
4. Attention to details. Best recommendations may fail because of concrete ‘minimal’ details like lack of explanation, lack of written prescriptions and insufficient patient´s resources. Principles of patient safety and ethics should be included any time we deal with patients with cancer-related pain.

## Conclusion

This article underscores the pervasive prevalence and detrimental impact of cancer-related pain, particularly accentuating global disparities in its management due to resource limitations and policies across countries. It highlights multidimensional barriers hindering effective pain control, including cultural beliefs and attitudes, insufficient training and regulatory constraints. Emphasising the pivotal role of compassionate care, it advocates for tailored approaches to pain management based on comprehensive assessment and individualised treatment plans. Integrated care involving pharmacological and non-pharmacological interventions by interdisciplinary teams is deemed essential, alongside attention to detail, patient safety and ethical considerations. Concerted efforts are needed to address disparities and barriers, and adopt effective approaches to alleviate unnecessary suffering and enhance the quality of life for cancer patients globally.

## Conflicts of interest

No competing interests are declared by the authors.

## Funding

The review was done without any financial support.

## Figures and Tables

**Table 1.** Pain classification according to neural mechanisms and descriptors.

Type		Neural mechanism	Example	Characteristics (descriptors)
Nociceptive	Visceral	Stimulation of pain receptors on normal sensory nerve endings	Spleen or liver enlargement, capsule distention Abdominal pain due to peritoneal invasion	Pressure, colic or cramping, squeezing, or aching. Diffuse and poorly defined, referred to distant sites. Associated with autonomic response (pallor, sweating, nausea, heart rate, anxiety.
	Somatic		Bone pain due to bone marrow invasion Mucositis	Oppressive, constant, throbbing, aching or gnawing, usually localized.
Neuropathic	Peripheral	Lesion or disease (injury, infiltration or compression) of the somatosensory nervous system which lead to altered or disordered transmission, sensitization or autonomous ectopic activity.	Plexopathy by tumor infiltration or destruction	Burning, pricking, pins and needles, tingling, shooting, lancinating, stabbing, or freezing. Paroxysmal pain. Evoked pain. Neuroanatomical distribution. Sensory symptoms or signs present. Hypersensitivity: Allodynia, hyperalgesia. Hypoesthesia: Numbness. Hyposensitivity. Paresthesia/dysesthesia, etc. Muscle wasting, fasciculations. Sympathetic component, sweating, increased skin temperature.
	Central		Spinal cord compression by tumor	
	Mixed		Central sensitization due to unrelieved peripheral neuropathic pain	
	Sympathetically maintained	Dysfunction of sympathetic system	Chronic regional pain syndrome following fracture or other trauma. Acute herpetic pain.	
Mixed	Neuropathic + somatic (+/nociplastic*)		Bone metastasis	A combination of both etiologies

‘*Pain that arises from altered nociception despite no clear evidence of actual or threatened tissue damage causing the activation of peripheral nociceptors or evidence for disease or lesion of the somatosensory system causing the pain’ IASP Terminology. Washington DC, USA: International Association for the Study of Pain; c2018

**Table 2.** SOCRATES and OPQRSTU mnemonics.

Site Onset Characteristics Radiation Associated symptoms Timing Exacerbating or alleviating factors Severity	Localization, where is the pain? When did it start? What does it feel like? How would you describe you pain? Does it spread? Would you mention any other symptoms related with your pain? Is it continuous or intermittent? Does it appear at a specific moment during the day? What makes it worse or reliefs you pain? Response to prior pain therapies? Could you rate your pain from 0 to 10, with 0 being no pain and 10 being the worst pain ever? How severe is it?’ ‘How much does it affect your life?*
Onset Provocation/palliation Quality Region Severity Timing/treatment Understanding	When did it start? What makes it worse or better? Is there anything that triggers your pain? What does it feel like? Can you describe you pain? Is it burning, is it cramping? Localization. Where is the pain? How much does it hurt? Could you rate it in a scale from 0 to 10? Is it constant or intermittent? How long does it last? Has any intervention or treatment helped? What do you think is causing the pain? How does it interfere with daily living?

**Table 3.** Factors that influence pain threshold.

Negative factors (Decrease pain threshold and tend to increase pain perception)	Positive factors (Increase pain threshold and tend to decrease pain perception)
Anger Fatigue, disability Anxiety Depression Boredom Discomfort, fear Insomnia Grieving Lack of understanding about condition Mental isolation, social abandonment	Acceptance Relief of other symptoms Reduction in anxiety, relaxation Good mood Creative activities Proper sleep Solidarity Feelings of support and compassion Situation understanding Companionship

**Table 4.** Summary of interventional procedures for cancer pain management [120].

Type of intervention or procedure	Primary tumor or metastatic site indications
Nerve blocks *Peripheral nerves* Paravertebral Interescalene. *Plexus nerve* Celiac Superior hypogastric Ganglion impar	Chest-wall pain after mastectomy Upper-extremity pain after surgical repair of pathologic fractures and neuropathy from brachial plexoplathy Right-upper-quadrant and epigastric pain from pancreaticobiliary malignancies Pelvic pain from gynecologic and urologic malignancies Perineal and rectal pain from anorectal and vulvar malignancies
Implantable catheters and neuromodulation Intraspinal drug delivery Spinal cord stimulation Dorsal root ganglion stimulation	Visceral pain from abdominal malignancies, neuropathic pain for lower extremities, and intractable back pain from metastases
Vertebral augmentation Vertebroplasty Kyphoplasty Back pain from spine metastases and vertebral fractures Ablation procedures Radiofrequency ablation Cryoablation Microwave ablation Magnetic resonance imaging-guided focused-ultrasound surgery	Back pain from spine metastases and vertebral fractures Pain from metastatic bone and soft-tissues sites
Transarterial embolization	Pain from hypervascular bone metastases

## References

[1] Van denBeuken-van Everdingen MH, Hochstenbach LM, Joosten EA (2016). Update on prevalence of pain in patients with cancer: systematic review and meta-analysis. J Pain Symptom Manag.

[2] van den Beuken-van Everdingen MH, Rijke JM, Kessels AG (2007). Prevalence of pain in patients with cancer: a systematic review of the past 40 years. Ann Oncol.

[3] Pachman DR, Barton DL, Swetz KM (2012). Troublesome symptoms in cancer survivors: fatigue, insomnia, neuropathy, and pain. J Clin Oncol.

[4] Portenoy RK, Lesage P (1999). Management of cancer pain. Lancet.

[5] Breivik H, Cherny N, Collett B (2009). Cancer-related pain: a pan-European survey of prevalence, treatment, and patient attitudes. Ann Oncol.

[6] Quinten C, Coens C, Mauer M (2009). Baseline quality of life as a prognostic indicator of survival: a meta-analysis of individual patient data from EORTC clinical trials. Lancet Oncol.

[7] Beck SL, Towsley GL, Berry PH (2010). Core aspects of satisfaction with pain management: cancer patients’ perspectives. J Pain Symptom Manage.

[8] Basch E, Deal AM, Kris MG (2016). Symptom monitoring with patient-reported outcomes during routine cancer treatment: a randomized controlled trial. J Clin Oncol.

[9] Boland JW, Allgar V, Boland EG (2020). The relationship between pain, analgesics and survival in patients with advanced cancer; a secondary data analysis of the international European palliative care cancer symptom study. Eur J Clin Pharmacol.

[10] Wien PJ, Wee B, Moore RA (2016). Oral morphine for cancer pain. Cochrane Database Syst Rev.

[11] Ventafridda V, Tamburini M, Caraceni A (1987). A validation study of the WHO method for cancer pain relief. Cancer.

[12] Reddy A, Cruz M, Rodriguez EM (2014). Patterns of storage, use, and disposal of opioids among cancer outpatients. Oncologist.

[13] Roberto A, Greco MT, Uggeri S (2022). Living systematic review to assess the analgesic undertreatment in cancer patients. Pain Pract.

[14] Deandrea S, Montanari M, Moja L (2008). Prevalence of undertreatment in cancer pain: a review of published literature. Ann Oncol.

[15] Scarborough BM, Smith CB (2018). Optimal pain management for patients with cancer in the modern era. CA Cancer J Clin.

[16] Silbermann M, Calimag MM, Eisenberg E (2022). Evaluating pain management practices for cancer patients among health professionals: a global survey. J Palliat Med.

[17] León MX, Sánchez-Cárdenas MA, RodríguezCampos LF (2022). Availability and accessibility of opioids for pain and palliative care in Colombia: a survey study. Colomb J Anesthesiol.

[18] Lohman D, Schleifer R, Amon JJ (2010). Access to pain treatment as a human right. BMC Med.

[19] Globocan (2020). Cancer today: world fact sheet. http://gco.iarc.f.

[20] World Cancer Research Foundation International (2020). https://www.wcrf.org/cancer-trends/worldwide-cancer-data/World.

[21] Strasser-Weippl K, Chavarri-Guerra Y, Villarreal-Garza C (2015). Progress and remaining challenges for cancer control in Latin America and the Caribbean. Lancet Oncol.

[22] Mestdagh F, Steyaert A, Lavand'homme P (2023). Cancer pain management: a narrative review of current concepts, strategies, and techniques. Curr Oncol.

[23] Sheinfeld Gorin S, Krebs P, Badr H (2012). Meta-analysis of psychosocial interventions to reduce pain in patients with cancer. J Clin Oncol.

[24] Snijders RAH, Brom L, Theunissen M (2023). Update on prevalence of pain in patients with cancer: a systematic literature review and meta-analysis. Cancers.

[25] Nijs J, Roose E, Lahousse A (2021). Pain and opioid use in cancer survivors: a practical guide to account for perceived injustice. Pain Physician.

[26] Caraceni A, Portenoy RK (1999). An international survey of cancer pain characteristics and syndromes: IASP task force on cancer pain – International Association for the Study of Pain. Pain.

[27] Van den Beuken-van Everdingen MHJ, Van Kuijk SMJ, Janssen DJA (2018). Treatment of pain in cancer: towards personalised medicine. Cancers.

[28] (2018). WHO Guidelines for the Pharmacological and Radiotherapeutic Management of Cancer Pain in Adults and Adolescents.

[29] Petroianu GA, Aloum L, Adem A (2023). Neuropathic pain: mechanisms and therapeutic strategies. Front Cell Dev Biol.

[30] Freynhagen R, Arevalo Parada H, Calderon-Ospina CA (2019). Current understanding of the mixed pain concept: a brief narrative review. Curr Med Res Opin.

[31] Baron R, Binder A, Wasner G (2018). Neuropathic pain: diagnosis, pathophysiological mechanisms, and treatment. Lancet Neurol.

[32] Twycross R, Wilcock A, Stark Toller C (2021). 6th edn (London: Pharmaceuticals Press). Introducing Palliative Care.

[33] Yoon SY, Oh J (2018). Neuropathic cancer pain: prevalence, pathophysiology, and management. Korean J Intern Med.

[34] Fallon MT (2013). Neuropathic pain in cancer. Br J Anaesth.

[35] Mulvey MR, Boland EG, Bouhassira D (2017). Neuropathic pain in cancer: systematic review, performance of screening tools and analysis of symptom profiles. Br J Anaesth.

[36] Yoneda T, Hiasa M, Nagata Y (2015). Contribution of acidic extracellular microenvironment of cancercolonized bone to bone pain. Biochim Biophys Acta.

[37] Fallon M, Giusti R, Aielli F (2018). Management of cancer pain in adult patients: ESMO clinical practice guidelines. Ann Oncol.

[38] Finnerup NB, Haroutounian S, Kamerman P (2016). Neuropathic pain: an updated grading system for research and clinical practice. Pain.

[39] Bouhassira D, Luporsi E, Krakowski I (2017). Prevalence and incidence of chronic pain with or without neuropathic characteristics in patients with cancer. Pain.

[40] Ngamkham S, Holden JE, Wilkie DJ (2011). Differences in pain location, intensity, and quality by pain pattern in outpatients with cancer. Cancer Nurs.

[41] Mercadante S (2015). Breakthrough pain in cancer patients: prevalence, mechanisms and treatment options. Curr Opin Anaesthesiol.

[42] LeBlanc TW, Howie LI, Abernethy AP, Alberts DS, LluriaPrevatt M, Kha S (2016). Breakthrough cancer pain. Supportive Cancer Care.

[43] Brant JM, Rodgers BB, Gallagher E (2017). Breakthrough cancer pain: a systematic review of pharmacologic management. Clin J Oncol Nurs.

[44] Bennett MI, Kaasa S, Barke A (2019). IASP taskforce for the classification of chronic pain. The IASP classification of chronic pain for ICD-11: chronic cancer-related pain. Pain.

[45] Twycross R (1984). Control of pain. J R Coll Physicians London.

[46] Brant JM, Stringer LH (2018). Cancer pain. Cancer Nursing: Principles and Practice.

[47] Fink RM, Gallagher E (2019). Cancer pain assessment and measurement. Semin Oncol Nurs.

[48] Fink RM, Brant JM (2018). Complex cancer pain assessment. Hematol Oncol Clin North Am.

[49] Cherny NI, Marie FT, Stein K (2021). Chronic cancer pain syndromes. Oxford Textbook of Palliative Medicine.

[50] Dhanju S, Kennedy SH, Abbey S (2019). The impact of comorbid pain and depression in the United States: results from a nationally representative survey. Scand J Pain.

[51] Hooten WM (2016). Chronic pain and mental health disorders: shared neural mechanisms, epidemiology, and treatment. Mayo Clin Proc.

[52] Linton SJ, Shaw WS (2011). Impact of psychological factors in the experience of pain. Phys Ther.

[53] Zaza C, Baine N (2002). Cancer pain and psychosocial factors. J Pain Symptom Manage.

[54] Forte AJ, Guliyeva G, McLeod H (2022). The impact of optimism on cancer-related and postsurgical cancer pain: a systematic review. JPSM.

[55] Fink R (2000). Pain assessment: the cornerstone to optimal pain management. Proc (Bayl Univ Med Cent).

[56] National Comprehensive Cancer Network (2016). NCCN Clinical Practice Guidelines in Oncology: Adult Cancer Pain.

[57] Herr K, Coyne PJ, McCaffery M (2011). Pain assessment in the patient unable to self-report: position statement with clinical practice recommendations. Pain Manag Nurs.

[58] Zalon ML (1995). Pain management in struction in nursing curricula. J Nurs Educ.

[59] Hui D, Bruera E (2014). A personalized approach to assessing and managing pain in patients with cancer. J Clin Oncol.

[60] Shkodra M, Brunelli C, Zecca E (2022). Cancer pain: results of a prospective study on prognostic indicators of pain intensity including pain syndromes assessment. Palliat Med.

[61] Scher C, Meador L, Van Cleave JH (2018). Moving beyond pain as the fifth vital sign and patient satisfaction scores to improve pain care in the 21st century. Pain Manag Nurs.

[62] Ngamkham S, Vincent C, Finnegan L (2012). The McGill pain questionnaire as a multidimensional measure in people with cancer: an integrative review. Pain Manag Nurs.

[63] McGuire D, Snow Kaiser K, Haisfield-Wolfe ME (2016). Pain assessment in non-communicative adult palliative care patients. Nurs Clin North Am.

[64] Caraceni A, Cherny N, Fainsinger R (2002). Pain measurement tools and methods in clinical research in palliative care: recommendations of an expert working group of the European Association of Palliative Care. J Pain Symptom Manage.

[65] Jensen MP (2003). The validity and reliability of pain measures in adults with cancer. J Pain.

[66] Swarm RA, Paice JA, Anghelescu DL (2019). Adult cancer pain, version 3.2019. J Natl Compr Canc Netw.

[67] Fillingim RB, Loeser JD, Baron R (2016). Assessment of chronic pain: domains, methods, and mechanisms. J Pain.

[68] Mystakidou K, Parpa E, Tsilika E (2002). Greek McGill pain questionnaire: validation and utility in cancer patients. J Pain Symptom Manage.

[69] Caraceni A, Mendoza TR, Mencaglia E (1996). A validation study of an Italian version of the brief pain inventory. Pain.

[70] Bennett MI, Attal N, Backonja MM (2007). Using screening tools to identify neuropathic pain. Pain.

[71] Haussleiter IS, Richter H, Scherens A (2008). NeuroQuick–a novel bedside test for small fiber neuropathy?. Eur J Pain.

[72] Cruccu G, Truini A (2009). Tools for assessing neuropathic pain. PLoS Med.

[73] Bennett M (2001). The LANSS Pain Scale: the leeds assessment of neuropathic symptoms and signs. Pain.

[74] Giannitrapani KF, Day RT, Azarfar A (2019). What do providers want from a pain screening measure used in daily practice?. Pain Med.

[75] Scher C, Petti E, Meador L (2020). Multidimensional pain assessment tools for ambulatory and inpatient nursing practice. Pain Manag Nurs.

[76] Payen JF, Bru O, Bosson JL (2001). Assessing pain in critically ill sedated patients by using a behavioral pain scale. Crit Care Med.

[77] Aïssaoui Y, Zeggwagh AA, Zekraoui A (2005). Validation of a behavioral pain scale in critically ill, sedated, and mechanically ventilated patients. Anesth Analg.

[78] Feldt KS (2000). The checklist of nonverbal pain indicators (CNPI). Pain Manag Nurs.

[79] McGuire DB, Kaiser KS, Soeken K (2011). Measuring pain in non-communicative palliative care patients in an acute care setting: psychometric evaluation of the multidimensional objective pain assessment tool (MOPAT). J Pain Sympt Manag.

[80] Lefebvre-Chapiro S (2001). The DOLOPLUS 2 scale – evaluating pain in the elderly. Eur J Palliat Care.

[81] Warden V, Hurley AC, Volicer L (2003). Development and psychometric evaluation of the Pain Assessment in Advanced Dementia (PAINAD) scale. J Am Med Dir Assoc.

[82] Hachem GE, Rocha FO, Pepersack T (2019). Advances in pain management for older patients with cancer. ecancer.

[83] Novy DM, Aigner CJ (2014). The biopsychosocial model in cancer pain. Curr Opin Support Palliat Care.

[84] Khatibi A, Sharpe L, Jafari H (2014). Interpretation biases in chronic pain patients: an incidental learning task. Eur J Pain.

[85] Jensen MP, Romano JM, Turner JA (1999). Patient beliefs predict patient functioning: further support for a cognitive-behavioural model of chronic pain. Pain.

[86] Khan RS, Ahmed K, Blakewayt E (2011). Catastrophizing: a predictive factor for postoperative pain. Am J Surg.

[87] Galloway SK, Baker M, Giglio P (2012). Depression and anxiety symptoms relate to distinct components of pain experience among patients with breast cancer. Pain Res Treat.

[88] Robinson S, Kisanne D, Brooker J (2015). A systematic review of the demoralization syndrome in individuals with progressive disease and cancer: a decade of research. JPSM.

[89] Delgado-Guay M, Parsons HA, Li Z (2009). Symptom distress in advanced cancer patients with anxiety and depression in the palliative care setting. Support Care Cancer.

[90] Hui D, Cruz M, Thorney S (2011). The frequency and correlates of spiritual distress among patients with advanced cancer admitted to an acute palliative care unit. Am J Hosp Palliat Care.

[91] Ellingsen DM, Isenburg K, Jung C (2023). Brain-to-brain mechanisms underlying pain empathy and social modulation of pain in the patient-clinician interaction. Proc Nat Acad Sci.

[92] Colvin L, Forbes K, Fallon M (2006). Difficult pain. BMJ.

[93] Ciucă A, Băban A (2017). Psychological factors and psychosocial interventions for cancer related pain. Rom J Intern Med.

[94] MacAdam D (2015). An initial assessment of suffering in terminal illness. Palliat Med.

[95] Lionis C (2015). Why and how is compassion necessary to provide good healthcare? Comments from an academic physician. Int J Health Policy Manag.

[96] Brito-Pons G, Librada-Flores S (2018). Compassion in palliative care: a review. Curr Opin Support Palliat Care.

[97] Malenfant S, Jaggi P, Hayden KA (2022). Compassion in healthcare: an updated scoping review of the literature. BMC Palliat Care.

[98] Perez-Bret E, Altisent R, Rocafort J (2016). Definition of compassion in healthcare: a systematic literature review. Int J Palliat Nurs.

[99] Brito G, Waibel A, Rosenberg E (2019). Compassion cultivation trainning: programme description. Research and potential benefits. The Power of Compassion.

[100] Sandhu H, Booth K, Furlan A (2023). Reducing opioid use for chronic pain with a group-based intervention. JAMA.

[101] Farrar JT, Portenoy RK, Berlin JA (2000). Defining the clinically important difference in pain outcome measures. Pain.

[102] Farrar JT, Young JP, LaMoreaux L (2001). Clinical importance of changes in chronic pain intensity measured on an 11- point numerical pain rating scale. Pain.

[103] Mills S, Torrance N, Smith BH (2016). Identification and management of chronic pain in primary care: a review. Curr Psychiatry Rep.

[104] Zhang M, Zhang Y, Kong Y (2019). Interaction between social pain and physical pain. Brain Sci Adv.

[105] Street RL, Makoul G, Arora NK (2009). How does communication heal? Pathways linking clinician-patient communication to health outcomes. Patient Educ Couns.

[106] Gallagher E, Rogers BB, Brant JM (2017). Cancer-related pain assessment: monitoring the effectiveness of interventions. Clin J Oncol Nurs.

[107] Qaseem A, Wilt TJ, McLean RM (2017). Noninvasive treatments for acute, subacute, and chronic low back pain: a clinical practice guideline from the American College of Physicians. Ann Intern Med.

[108] Chou R, Deyo R, Friedly J (2017). Nonpharmacologic therapies for low back pain: a systematic review for an American College of Physicians Clinical Practice Guideline. Ann Intern Med.

[109] Maindet C, Burnod A, Minello C (2019). Strategies of complementary and integrative therapies in cancer-related pain –attaining exhaustive cancer pain management. Support Care Cancer.

[110] Katta M, Valisekka S, Katamreddy T J Oncol Pharm Pract.

[111] Lee M, Wang X, Liu C (2018). Outcome literature review of integrative body–mind–spirit practices for mental health conditions. Soc Work Res.

[112] Deng G (2019). Integrative medicine therapies for pain management in cancer patients. Cancer J.

[113] Lyman GH, Greenlee H, Bohlke K (2018). Integrative therapies during and after breast cancer treatment: ASCO endorsement of the SIO clinical practice guideline. J Clin Oncol.

[114] Greenlee H, DuPont-Reyes MJ, Balneaves LG (2017). Clinical practice guidelines on the evidence-based use of integrative therapies during and after breast cancer treatment. CA Cancer J Clin.

[115] Syrjala KL, Donaldson GW, Davis MW (1995). Relaxation and imagery and cognitive-behavioral training reduce pain during cancer treatment: a controlled clinical trial. Pain.

[116] Keefe FJ, Abernethy AP, Campbell LC (2005). Psychological approaches to understanding and treating disease-related pain. Annu Rev Psychol.

[117] Puchalski C, Ferrell B, Virani R (2009). Improving the quality of spiritual care as a dimension of palliative care: the report of the Consensus Conference. J Palliat Med.

[118] World Health Organization (1996). Cancer Pain Relief, With a Guide to Opioid Availability.

[119] Huang R, Jiang L, Cao Y (2019). Comparative efficacy of therapeutics for chronic cancer pain: a Bayesian network meta-analysis. J Clin Oncol.

[120] Samala RV, Lagman RL, Najafi S (2021). Frequently asked questions about managing cancer pain: an update. Cleve Clin J Med.

[121] Strawson J (2018). Nonsteroidal anti-inflammatory drugs and cancer pain. Curr Opin Support Palliat Care.

[122] Magee DJ, Jhanji S, Poulogiannis P (2019). Nonsteroidal anti-inflammatory drugs and pain in cancer patients: a systematic review and reappraisal of the evidence. Br J Anaesth.

[123] Leiva-Vásquez O, Letelier LM, Rojas L (2023). Is acetaminophen beneficial in patients with cancer pain who are on strong opioids? a randomized controlled trial. J Pain Symptom Manage.

[124] Eusebi LH, Rabitti S, Artesiani ML (2017). Proton pump inhibitors: risks of longterm use. J Gastroenterol Hepatol.

[125] Wiffen PJ, Derry S, Moore RA (2017). Oral paracetamol (acetaminophen) for cancer pain. Cochrane Database Syst Rev.

[126] Caraceni A, Hanks G, Kaasa S (2012). Use of opioid analgesics in the treatment of cancer pain: evidence-based recommendations from the EAPC. Lancet Oncol.

[127] Altyar A, Kordi L, Skrepnek G (2015). Clinical and economic characteristics of emergency department visits due to acetaminophen toxicity in the USA. BMJ Open.

[128] Leiva O, Castellano J, Letelier LM (2022). Randomized double-blind controlled trial to assess the efficacy of intravenous acetaminophen associated with strong opioids in the treatment of acute pain in adult cancer patients: study protocol. Trials.

[129] Mawatari H, Shinjo T, Morita T (2022). Revision of pharmacological treatment recommendations for cancer pain: clinical guidelines from the Japanese Society of Palliative Medicine. J Palliat Med.

[130] Paice JA, Bohlke K, Barton D (2022). Use of opioids for adults with pain from cancer or cancer treatment: ASCO guideline. J Clin Oncol.

[131] Bramati S, Bruera E (2022). The end of the second step of the World Health Organization analgesic ladder?. Ann Oncol.

[132] Fallon M, Dierberger K, Leng M (2022). An international, open-label, randomised trial comparing a two-step approach versus the standard three-step approach of the WHO analgesic ladder in patients with cancer. Ann Oncol.

[133] Mercadante S (2007). Opioid titration in cancer pain: a critical review. Eur J Pain.

[134] Mercadante S (2022). Cancer pain treatment strategies in patients with vancer. Drugs.

[135] Cherny N, Ripamonti C, Pereira J (2001). Strategies to manage the adverse effects of oral morphine: an evidence-based report. J Clin Oncol.

[136] Schmidt-Hansen M, Bromham N, Taubert M (2015). Buprenorphine for treating cancer pain. Cochrane Database Syst Rev.

[137] Virgen C, Kelkar N, Tran A (2022). Pharmacological management of cancer pain: novel therapeutics. Biomed Pharmacother.

[138] Roy PJ, Weltman M, Dember LM (2020). Pain management in patients with chronic kidney disease and end-stage kidney disease. Curr Opin Nephrol Hypertens.

[139] Pergolizzi J, Böger RH, Budd K (2008). Opioids and the management of chronic severe pain in the elderly: consensus statement of an International Expert Panel with focus on the six clinically most often used World Health Organization Step III opioids (buprenorphine, fentanyl, hydromorphone, methadone, morphine, oxycodone). Pain Pract.

[140] Pergolizzi JV, Raffa RB (2019). Safety and efficacy of the unique opioid buprenorphine for the treatment of chronic pain. J Pain Res.

[141] Naing C, Yeoh PN, Aung K (2014). A meta-analysis of efficacy and tolerability of buprenorphine for the relief of cancer pain. Springer Plus.

[142] Al-Tawil N, Odar-Cederlof I, Berggren AC (2013). Pharmacokinetics of transdermal buprenorphine patch in the elderly. Eur J Clin Pharmacol.

[143] Mercadante S, Adile C, Ferrera P (2022). Methadone as first-line opioid for the management of cancer pain. Oncologist.

[144] Wiffen PJ, Wee B, Moore RA (2016). Oral morphine for cancer pain. Cochrane Database Syst Rev.

[145] Ding H, Song Y, Xin W (2022). Methadone switching for refractory cancer pain. BMC Palliat Care.

[146] Mercadante S, Bruera E (2016). Opioid switching in cancer pain: from the beginning to nowadays. Crit Rev Oncol Hematol.

[147] Mercadante S, Bruera E (2018). Methadone as a first-line opioid in cancer pain management: a systematic review. J Pain Symptom Manage.

[148] Mammana G, Bertolino M, Bruera E (2021). First-line methadone for cancer pain: titration time analysis. Support Care Cancer.

[149] Mercadante S (2010). Intravenous morphine for management of cancer pain. Lancet Oncol.

[150] Bennett MI (2011). Effectiveness of antiepileptic or antidepressant drugs when added to opioids for cancer pain: systematic review. Palliat Med.

[151] Leppert W, Buss T (2012). The role of corticosteroids in the treatment of pain in cancer patients. Curr Pain Headache Rep.

[152] Haywood A, Good P, Khan S (2015). Corticosteroids for the management of cancer-related pain in adults. Cochrane Database Syst Rev.

[153] Swarm RA, Abernethy AP, Anghelescu DL (2013). National comprehensive cancer network. Adult cancer pain. J Natl Compr Canc Netw.

[154] Kurita GP, Sjøgren P, Klepstad P (2019). Interventional techniques to management of cancer-related pain: clinical and critical aspects. Cancers (Basel).

[155] Okuyama M, Shibata T, Morita T (2002). A comparison of intraoperative celiac plexus block with pharmacological therapy as a treatment for pain of unresectable pancreatic cancer. J Hepatobiliary Pancreat Surg.

[156] Wise JM, Carone M, Paquin SC (2011). Randomized, double-blind, controlled trial of early endoscopic ultrasound– guided celiac plexus neurolysis to prevent pain progression in patients with newly diagnosed, painful, inoperable pancreatic cancer. J Clin Oncol.

[157] Mercadante S, Klepstad P, Kurita GP (2015). European palliative care research collaborative (EPCRC). Sympathetic blocks for visceral cancer pain management: a systematic review and EAPC recommendations. Crit Rev Oncol Hematol.

[158] Mercadante S, Klepstad P, Kurita GP (2016). Minimally invasive procedures for the management of vertebral bone pain due to cancer: the EAPC recommendations. Acta Oncol.

[159] Hochberg U, Minerbi A, Boucher LM (2020). Interventional pain management for cancer pain: an analysis of outcomes and predictors of clinical response. Pain Physician.

[160] Hochberg U, Ingelmo P, Solé E (2023). Early interventional treatments for patients with cancer pain: a narrative review. J Pain Res.

[161] Sazuk S, Koitabashi T (2020). Tapentadol is effective in the management of moderate‑to‑severe cancer‑related pain in opioid‑naïve and opioid‑tolerant patients: a retrospective study. J Anesth.

[162] Mercadante S, Porzio G, Ferrera P (2012). Tapentadol in cancer pain management: a prospective open-label study. Curr Med Res Opin.

[163] Walker JM, Huang SM (2002). Cannabinoid analgesia. Pharmacol Ther.

[164] Lichtman AH, Lux EA, McQuade R (2018). Results of a double-blind, randomized, placebo-controlled study of nabiximols oromucosal spray as an adjunctive therapy in advanced cancer patients with chronic uncontrolled pain. J Pain Symptom Manag.

[165] Häuser W, Welsch P, Klose P (2019). Efficacy, tolerability and safety of cannabis-based medicines for cancer pain: a systematic review with meta-analysis of randomised controlled trials. Schmerz.

[166] Johnson JR, Burnell-Nugent M, Lossignol D (2010). Multicenter, double-blind, randomized, placebo-controlled, parallel-group study of the efficacy, safety, and tolerability of THC:CBD extract and THC extract in patients with intractable cancer-related pain. J Pain Symptom Manag.

[167] Portenoy RK, Ganae-Motan ED, Allende S (2012). Nabiximols for opioid-treated cancer patients with poorly-controlled chronic pain: a randomized, placebo-controlled, graded-dose trial. J Pain.

[168] Mittal SO, Jabbari B (2020). Botulinum neurotoxins and cancer – a review of the literature. Toxins.

[169] Grenda T, Grenda A, Krawczyk P (2022). Botulinum toxin in cancer therapy-current perspectives and limitations. Appl Microbiol Biotechnol.

[170] Stopeck AT, Lipton A, Body JJ (2010). Denosumab compared with zoledronic acid for the treatment of bone metastases in patients with advanced breast cancer: a randomized, double-blind study. J Clin Oncol.

[171] Fizazi K, Carducci M, Smith M (2011). Denosumab versus zoledronic acid for treatment of bone metastases in men with castration-resistant prostate cancer: a randomised, double-blind study. Lancet (London, England).

[172] Dhabhar B (2022). Cancer treatment-induced bone loss: role of denosumab in nonmetastatic breast cancer. Breast Cancer.

[173] Farrer E, Dickman A (2022). New analgesics in cancer pain. Curr Opin Support Palliat Care.

[174] Gonzalez-Cano R, Ruiz-Cantero MC, Santos-Caballero Gómez-Navas M (2021). Tetrodotoxin, a potential drug for neuropathic and cancer pain relief?. Toxins.

[175] Hagen NA, Cantin L, Constant J (2017). Tetrodotoxin for moderate to severe cancer-related pain: a multicentre, randomized, double-blind, placebo-controlled, parallel-design trial. Pain Res Manag.

[176] Fallon MT, Storey DJ, Krishan A (2015). Cancer treatment-related neuropathic pain: proof of concept study with menthol–a TRPM8 agonist. Support Care Cancer.

[177] Wang ZY, Han QQ, Deng MY (2020). Lemairamin, isolated from the Zanthoxylum plants, alleviates pain hypersensitivity via spinal α7 nicotinic acetylcholine receptors. Biochem Biophys Res Commun.

[178] Scheff NN, Wall IM, Nicholson S (2022). Oral cancer induced TRPV1 sensitization is mediated by PAR2 signaling in primary afferent neurons innervating the cancer microenvironment. Sci Rep.

[179] Glare P, Aubrey K, Gulati A (2022). Pharmacologic management of persistent pain in cancer survivors. Drugs.

[180] Dzierżanowski T, Mercadante S (2022). Constipation in cancer patients – an update of clinical evidence. Curr Treat Options Oncol.

[181] Tsukuura H, Miyazaki M, Morita T (2018). Efficacy of prophylactic treatment for oxycodone-induced nausea and vomiting among patients with cancer pain (POINT): a randomized, placebo-controlled, double-blind trial. Oncologist.

[182] Hasselström J, Eriksson S, Persson A (1990). The metabolism and bioavailability of morphine in patients with severe liver cirrhosis. Br J Clin Pharmacol.

[183] OxyContin (Oxycodone Hydrochloride Extended-Release Tablets), for Oral Use. Stamford, Conn.: Purdue Pharma L.P.

[184] Soleimanpour H, Safari S, Shahsavari Nia K (2016). Opioid drugs in patients with liver disease: a systematic review. Hepat Mon.

[185] Ojeda A, Moreno LA (2014). Tratamiento del dolor en el paciente con cirrosis hepática [Pain management in patients with liver cirrhosis]. Gastroenterol Hepatol.

[186] Benson GD, Koff RS, Tolman KG (2005). The therapeutic use of acetaminophen in patients with liver disease. Am J Ther.

[187] Malespin MH (2018). Risk of nonsteroidal anti-inflammatory drugs and safety of acetaminophen in patients with advanced lider disease. Clin Liver Dis.

[188] Arroyo V, Ginés P, Rimola A (1986). Renal function abnormalities, prostaglandins, and effects of nonsteroidal anti-inflammatory drugs in cirrhosis with ascites. An overview with emphasis on pathogenesis. Am J Med.

[189] Beuken-van Everdingen Van den MHJ, De Rijke JM, Kessels AG (2007). Prevalence of pain in patients with cancer: a systematic review of the past 40 years. Ann Onc.

[190] Kwon JH (2014). Overcoming barriers in cancer pain management. J Clin Oncol.

[191] Krakauer EL, Wenk R, Buitrago R (2010). Opioid inaccessibility and its human consequences: reports from the field. J Pain Palliat Care Pharmacother.

[192] Vahos J, Rojas-Cortés R, Daza D (2023). Barriers of access to opioid medicines within the context of palliative care in Latin America: the perception of health professionals. J Palliat Med.

[193] Liñeiro MG, Santos Garcia JB, Narváez Tamayo MA (2023). Map of pain education in Latin America: current state and perspectives. Pain Manag.

[194] Lincoln SB, Soto-Perez-de-Celis E, Chavarri-Guerra Y (2019). Cancer pain management in Mexico. BMJ Support Palliat Care.

[195] Rico MA, Kraychete DC, Iskandar AJ (2016). Use of opioids in Latin America: the need of an evidence-based change. Pain Med.

[196] Sampaio R, Azevedo LF, Dias CC (2019). Portuguese version of the Medication Adherence Report Scale (MARS-9): validation in a population of chronic pain patients. J Eval Clin Pract.

[197] Garcia JBS, Lopez MPG, Barros GAM (2019). Latin American pain federation position paper on appropriate opioid use in pain management. Pain Rep.

[198] Mercadante S (2019). Potential strategies to combat the opioid crisis. Expert Opin Drug Saf.

[199] Dowell D, Jones C (2022). CDC clinical practice guideline for prescribing opioids for pain – United States,. MMWR Recomm Rep.

[200] Turk D, Monarch ES, Williams AD (2002). Cancer patients in pain: considerations for assessing the whole person. Hematol Oncol Clin North Am.

[201] Pasternak GW (2014). Opiate pharmacology and relief of pain (American Society of Clinical Oncology). J Clin Oncol.

